# Structural Investigation of Biologically Active Phenolic Compounds Isolated from European Tree Species

**DOI:** 10.3390/molecules14104147

**Published:** 2009-10-16

**Authors:** Izabela Redzynia, Natasza E. Ziółkowska, Wiesław R. Majzner, Stefan Willför, Rainer Sjöholm, Patrik Eklund, Grzegorz D. Bujacz

**Affiliations:** 1 Institute of Technical Biochemistry, Technical University of Łódź, ul. Stefanowskiego 4/10, 90-924 Łódź, Poland; E-Mails: izared@p.lodz.pl (I.R.); nataszaz@biochem.mpg.de (N.E.Z.); wmajzner@p.lodz.pl (W.R.M.); 2 Laboratory of Organic Chemistry, Abo Akademi University, Biskopsgatan 8, FI-20500 Abo, Finland; E-Mail: swillfor@abo.fi (S.W.); 3 Laboratory of Wood and Paper Chemistry, Abo Akademi University, Porthansgatan 3, FI-20500 Abo, Finland; E-Mails: rainer.sjoholm@abo.fi (R.S.); paeklund@abo.fi (P.E.)

**Keywords:** phenolic compounds, lignans, flavonoids, antioxidant potency, crystal structure, "host - guest" inclusion crystals

## Abstract

X-ray structures of two compounds isolated from wood knots of coniferous trees, namely dihydrokaempferol (3,5,8,13-tetrahydroxyflavanon) and lariciresinol (3,14-dimetoxy-7,10-epoxylignan-4,15,19-triol), are presented here. Diffraction data for the dihydrokaempferol crystals were collected on a CAD4 diffractometer and on a synchrotron for the lariciresinol crystal. The investigated compounds inhibit lipid peroxidation and lariciresinol is additionally a good scavenger of superoxide radicals. The structural data presented in this work provide a useful basis for designing more active compounds with potential use as antioxidants.

## Introduction

Large amounts of bioactive phenolic compounds are present in the wood knots of several tree species. The amount of lignans in the knots can be up to several hundred times larger than in the adjacent stemwood [1,2,3,4,5]. The amount of extractable phenolic compounds is on average around 15% (w/w) in *Picea abies*, while *Populus tremula* and *Abies balsamea* can contain considerable amounts of interesting polyphenols. Those phenolic compounds can be potentially used as antioxidants in food, pharmaceuticals, and natural biocides such as bactericides, pesticides and fungicides [6]. Additionally, lignans are of great interest in the search for antitumor agents and have potential as chemotherapeutics [7,8,9,10].

The phenolic compounds are extracted from wood knots and purified by chromatographic methods [11,12]. The extract obtained from heartwood, foliage, phloem, bark, and cork of several species is a good resource of natural phenolic antioxidants [13,14,15,16] but it contains a mixture of different phenolic and nonphenolic compounds in the form of both glycosides and free aglycones. Glycosylation is not desirable, since it affects the antioxidant properties of phenolic compounds [17]. In comparison, the hydrophilic compounds in knots contain mainly free aglycones of flavonoids and lignans [1,2,3,4,5,18].

**Figure 1:**
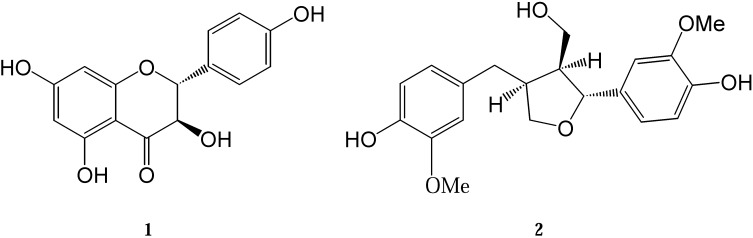
Structural formula of compounds **1** and **2**.

Wood knots of *P. tremula* and *A. balsamea* growing in Europe are rich in dihydrokaempferol (**1**) and lariciresinol (**2**) (Figure 1). Dihydrokaempferol - belonging to the flavanones group - shows a capacity to scavenge peroxyl radicals *in vitro*. The trapping capacity of that compound (expressed as the number of peroxy radicals in millimoles that are scavenged per gram of extract) is 0.78 mmol/g [19]. Lignans - among them lariciresinol - also inhibit lipid peroxidation. The trapping capacity of that compound in one of the test series was shown to be 7.3 mmol/g. In comparison, the trapping capacity of a well known antioxidant Trolox^®^ was reported as 6.8 mmol/g in the same test series [12]. Lariciresinol also reveals a capacity to scavenge superoxide radicals. Scavenging of superoxide radicals *in vitro* expressed as IC_50_ values (i.e., concentration of extract required for scavenging of 50% of the radicals) for this compound is 13 μg/L. A X-ray crystallography structural investigation of dihydrokaempferol and lariciresinol is presented in this paper.

## Results and Discussion

**Figure 2:**
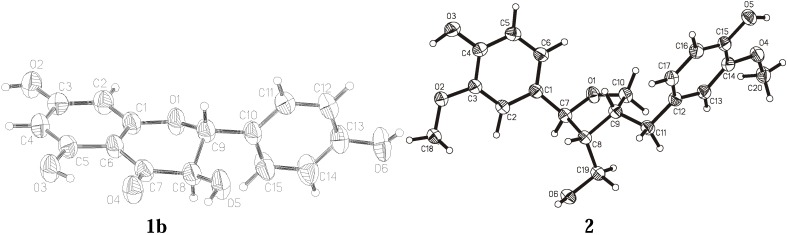
Thermal ellipsoidal view with the atom numbering scheme of the molecules of **1b** and **2**.

The crystal structures of two compounds isolated from the European tree species *P. tremula* and *A. balsamea* are presented here: dihydrokaempferol (3,5,8,13-tetrahydroxyflavanone) (**1**) and lariciresinol (3,14-dimethoxy-7,10-epoxylignan-4,15,19-triol) (**2**). Their chemical structures are depicted in Figure 1. The isolation and purification procedures of compound **1** and **2** and their spectroscopic characterization were described earlier [12,20]. Crystal data and experimental details for compound **1** and **2** are shown in Table 1. ORTEP views of the investigated molecules with the atom numbering schemes prepared using the program XP are shown in Figure 2 [21]. 

Two diffraction data sets were collected for **1** with different crystallization solvents and the structure was solved twice: **1a** with molecules of ethanol and **1b** with molecules of methanol trapped in the crystal lattices. Those are typical "host - guest" type inclusion crystals. The cell parameters *a*, *b* and *c* are similar for both **1a** and **1b**, which is typical for isostructural solvatomorphs [22]. The difference in cell volume is 34.5 A^3^, which well correlates with the volume of the two methylene groups that distinguish these two structures. The R_obs_ factor for **1a** is significantly higher than that of **1b** (Table 1), so only **1b** was further analyzed.

The analysis of bond lengths of compound **1** and **2** shows that their values do not differ significantly from typical values for compounds deposited in Cambridge Crystallographic Data Centre [23]. Elongation of bonds C7-C8, C8-C9, O1-C9 and C9-C10 observed in **1** is typical and caused by asymmetry of heterocyclic ring O1,C1,C6,C7,C8,C9. That ring adopts a conformation halfway between a half-chair and a sofa. In comparison, the five-membered ring of **2** is in a half-chair conformation. The asymmetry parameters indicating the lowest discrepancy from the dominant symmetry elements are shown in Table 2. The C-C and C-O bonds in the five-membered ring of **2** and also the bonds between carbon atoms (C7, C11) and *m*-methoxy-*p*-hydroxyphenyl groups of **2** are elongated. The aromatic ring of the hydroxyphenyl substituent can rotate around the C9-C10 bond in the molecule of **1**. Free rotation of the aromatic ring of the *m*-methoxy-*p*-hydroxyphenyl substituent in **2** can occur around only one bond (C1-C7), whilst the second substituent of that type can rotate around two bonds C9-C11 and C11-C12. The values of selected torsion angles of **1** and **2** are presented in Table 3.

**Table 1:** Crystal data and experimental details for compound **1** and **2**.

Compound		1a		1b		2	
Molecular formula		C_15_H_12_O_6_*CH_3_CH_2_OH		C_15_H_12_O_6_*CH_3_OH		C_20_H_24_O_6_	
Formula weight		334.31		320.29		360.39	
CCDC No.		719360		719361		719362	
Crystallographic system		triclinic		triclinic		monoclinic	
Space group		P1		P1		P2_1_	
a [Å]		7.617(5)		7.581(2)		10.718(6)	
b [Å]		10.349(3)		10.275(2)		5.656(3)	
c [Å]		11.488(3)		11.120(2)		14.264(8)	
α [^o^]		63.92(2)		65.28(3)			
β [^o^]		85.36(4)		81.80(3)		92.75(5)	
γ [^o^]		79.18(3)		76.61(3)			
V [Å^3^]		798.9(6)		764.4(3)		863.7(8)	
Z		2		2		2	
D_c_ [g/cm^3^]		1.390		1.392		1.386	
μ [mm^-1^]		0.918		0.936		0.102	
Crystal dimensions [mm]		0.60x0.40x0.02		0.56x0.12x0.1		1.00x0.06x0.02	
Radiation, λ (Å)		CuKα, 1.54178		CuKα, 1.54178		synchrotron, 0.80420	
*hkl* ranges:	*h* =	-9	0	0	9	-14	14
	*k* =	-12	12	-12	12	-6	6
	*l* =	-14	14	-13	13	-19	19
EAC correction:	min.	0.8867		0.9392		NA	
	max.	0.9933		0.9980			
	ave.	0.9294		0.9679			
No. of reflections:	unique	3545		3396		4342	
	with *I*>0σ(*I*)	3353		3210		3372	
	obs. with *I*>2σ(*I*)	2982		2982		4007	
No. of parameters refined		472		454		332	
*R*_obs_		0.0691		0.0430		0.0431	
*wR*_obs_		0.1871		0.1376		0.1137	
R_int_		0.0000		0.0000		0.0000	
S_obs_		1.098		1.094		1.051	

R_obs_=Σ||F_o_|-|F_c_||/Σ|F_o_|; *w*R_obs_=[Σ[*w*(F_o_^2^-F_c_^2^)^2^]/ [Σ[*w*(F_o_^2^)^2^]]^1/2^; R_int_=Σ|h_i_-h_eq_|/Σh_ave_ ; S_obs_=[Σ[*w*(F_o_^2^-F_c_^2^)^2^]/(n-p)]^1/2^, where n – no of reflections, p – no of parameters.

**Table 2:** Asymmetry parameters [24] for heteroatom rings for compound **1b** and **2**.

**1b**					
molecule	1	1’	molecule	1	1’
ΔC_s_^C6^=ΔC_s_^C9^	12.2(8)	12.4(8)	ΔC_2_^C1-C6^=ΔC_2_^C8-C9^	13.3(9)	16.6(9)
**2**					
ΔC_s_^C8^	11.2(3)		ΔC_2_^C8-C9^	4.6(3)	
ΔC_s_^C9^	17.8(3)		ΔC_2_^C9-C10^	40.2(3)	

1 and 1’ – molecules in the asymmetric unit.

**Table 3:** Selected torsion angles (°) for compounds **1b** and **2**.

**1b**												
molecule				1	1’	molecule				1		1’
C1	C2	C3	O2	-177.3(3)	-179.2(4)	C1	O1	C9	C10	172.7(3)	-179.7(3)	
O2	C3	C4	C5	176.5(3)	-179.7(4)	O5	C8	C9	C10	60.4(4)	54.0(4)	
C3	C4	C5	O3	-178.7(3)	179.1(3)	C7	C8	C9	C10	-176.4(3)	176.8(3)	
O3	C5	C6	C1	180.0(3)	-178.8(3)	O1	C9	C10	C15	-65.8(4)	-66.5(4)	
O3	C5	C6	C7	2.9(5)	4.4(5)	C8	C9	C10	C15	52.8(5)	53.1(5)	
C6	C7	C8	O5	160.4(3)	161.4(3)	O1	C9	C10	C11	118.9(4)	114.5(4)	
C5	C6	C7	O4	-7.3(6)	-7.6(6)	C8	C9	C10	C11	-122.4(4)	-125.9(4)	
O4	C7	C8	O5	-22.5(5)	-20.0(5)	O6	C13	C14	C15	179.4(5)	-179.5(4)	
O4	C7	C8	C9	-144.9(3)	-142.1(3)							
**2**												
C18	O2	C3	C2	0.4(2)		O1	C7	C8	C19	-88.0(1)		
C18	O2	C3	C4	179.8(1)		C1	C7	C8	C19	149.7(1)		
O2	C3	C4	O3	-0.1(2)		O1	C7	C8	C9	33.6(1)		
C2	C3	C4	O3	179.3(1)		C19	C8	C9	C11	-43.2(2)		
O2	C3	C4	C5	180.0(1)		C9	C11	C12	C17	84.7(2)		
O3	C4	C5	C6	-178.6(1)		C9	C11	C12	C13	-92.5(2)		
C10	O1	C7	C1	107.9(1)		C20	O4	C14	C13	-0.2(2)		
C10	O1	C7	C8	-15.4(1)		C20	O4	C14	C15	177.7(1)		
C6	C1	C7	O1	-19.2(2)		C12	C13	C14	O4	-179.6(1)		
C2	C1	C7	O1	162.0(1)		O4	C14	C15	O5	-1.1(2)		
C6	C1	C7	C8	99.3(2)		C7	C8	C19	O6	-67.7(2)		
C2	C1	C7	C8	-79.4(2)								

1 and 1’ – molecules in the asymmetric unit.

The values of dihedral angles between the planes of the rings of **1** and **2** are presented in Table 4. Plane 2 passing through the atoms of the hydroxyphenyl substituent is almost perpendicular to the plane of the heterocyclic ring in **1**.

**Table 4:** Dihedral angles between the planes passing through selected atoms for compounds **1b** and **2**.

1b			2	
Plane 1 C1, C2, C3, C4, C5, C6			Plane 1 C1, C2, C3, C4, C5, C6	
Plane 2 C10, C11, C12, C13, C14, C15			Plane 2 C12, C13, C14, C15, C16, C17	
Plane 3 C7, C8, C9			Plane 3 O1, C7, C9, C10	
Plane 4 O1, C8, C9			Plane 4 C7, C8, C9	
Plane 5 O1, C1, C6, C7			Plane 5 O1, C7, C10	
molecule	1	1’		
1 / 2	85.76(14)	87.22(16)	1 / 2	38.61(4)
1 / 3	38.63(38)	40.55(22)	1 / 3	86.53(5)
2 / 3	56.54(36)	52.61(25)	2 / 3	55.71(5)
1 / 4	44.27(39)	50.10(21)	1 / 4	68.13(7)
2 / 4	88.23(19)	89.68(23)	2 / 4	75.63(8)
3 / 4	58.58(42)	63.41(28)	3 / 4	36.70(10)
1 / 5	3.06(20)	3.02(18)	1 / 5	80.48(8)
2 / 5	88.57(20)	89.78(17)	2 / 5	61.55(8)
3 / 5	35.64(42)	37.53(28)	3 / 5	6.16(9)
4 / 5	45.21(38)	50.32(20)	4 / 5	32.72(12)

1 and 1’ – molecules in the asymmetric unit.

Similarly, Plane 1 (passing through the atoms of one of the *m*-methoxy-*p*-hydroxyphenyl group) is almost perpendicular to Plane 3 (passing through atoms O1, C7, C9, C10) in **2**. In comparison Plane 2 (passing through the atoms of the second *m*-methoxy-*p*-hydroxyphenyl group) is inclined to Plane 3 at an angle of 55.71(5)° in **2**. 

**Figure 3:**
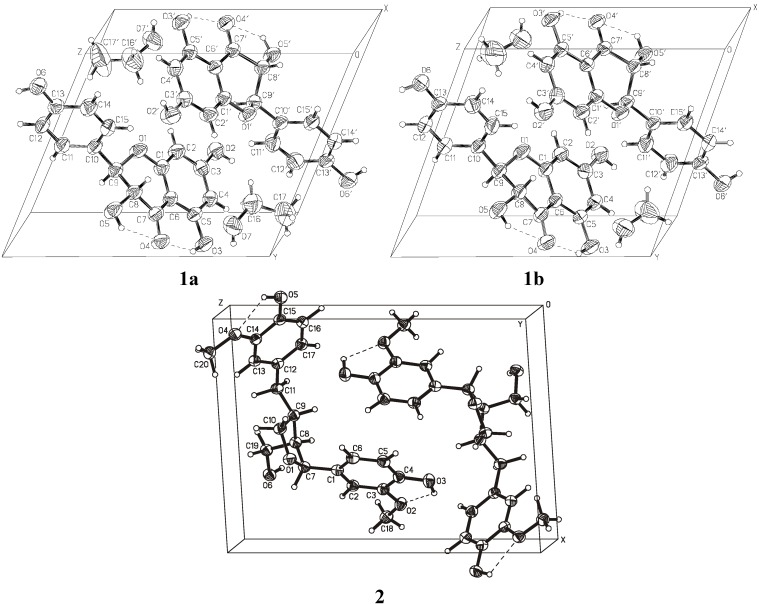
Crystal packing diagram for **1** and **2**.

**Figure 4:**
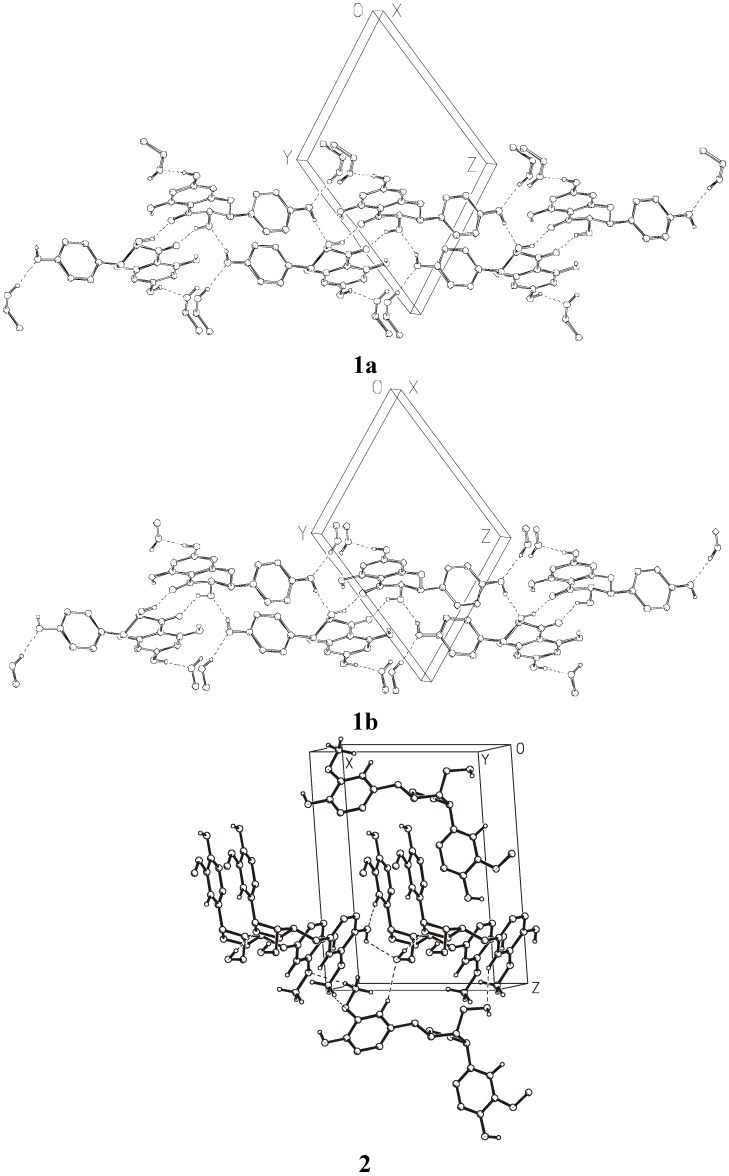
Intermolecular interactions in the crystal lattices of **1** and **2 (**hydrogens attached to carbon atoms are omitted for clarity).

Figure 3 shows the crystal packing and Figure 4 presents the intermolecular interactions in the crystal lattices of **1a**, **1b**, and **2**. The conformations of the molecules depend on the net of hydrogen bonds and π-stacking hydrophobic interactions influenced by the presence of solvent molecules. The hydrogen-bonding geometry for compounds **1** and **2** is shown in Table 5. Molecules of **1** create strong hydrogen bonds with the solvent molecules (**1a** with methanol and **1b** with ethanol, respectively). There are also two intramolecular hydrogen bonds O3−H3O···O4, O5−H5O···O4 and a few intermolecular hydrogen bonds in the crystal lattice of **1**. π-stacking interactions between aromatic rings of the molecules from neighboring unit cells are important factors determining the crystal packing of compound **2**. There are also two intramolecular hydrogen bonds O3−H3O···O2, O5−H5O···O4, and three intermolecular hydrogen bonds: O6−H6O···O1, O5−H5O···O6 in the crystal lattice of compound **2**.

**Table 5:** Hydrogen-bonding geometry (Å, °) (H···A not greater then 2.55Å) for **1** and **2**.

D―H···A	D―H	H···A	D···A	D―H···A
**1a**				
O3―H3O**···**O4	0.820(27)	1.926(30)	2.646(5)	146.0(36)
O5―H5O**···**O4	0.820(26)	2.265(28)	2.698(5)	113.4(28)
O3'―H3'O**···**O4'	0.820(14)	1.955(34)	2.656(5)	143.0(35)
O5'―H5'O**···**O4'	0.820(23)	2.231(20)	2.701(4)	116.7(23)
O5―H5O**···**O4' ^i^	0.820(26)	2.068(33)	2.767(5)	142.9(31)
O6―H6O**···**O5' ^ii^	0.820(36)	1.895(39)	2.698(6)	165.7(45)
O5'―H5'O**···**O4^ iii^	0.820(23)	2.088(20)	2.751(4)	137.7(25)
O6'―H6'O**···**O5^ iv^	0.820(43)	1.929(44)	2.663(7)	148.7(40)
O2―H2O**···**O7^ v^	0.820(42)	1.814(46)	2.616(6)	165.6(59)
O7―H7O**···**O6^vi^	0.820(18)	2.005(32)	2.796(7)	161.8(63)
O2'―H2'O**···**O7'^ vii^	0.820(19)	1.934(56)	2.630(6)	142.1(42)
O7'―H7'O**···**O6'^ viii^	0.820(16)	2.008(22)	2.817(7)	168.7(38)
C8―H8**···**O2^ vii^	0.980(8)	2.406(6)	3.133(7)	130.5(5)
**1b**				
O3―H3O**···**O4	0.820(18)	1.925(22)	2.646(4)	146.3(28)
O5―H5O**···**O4	0.820(23)	2.303(27)	2.689(4)	109.4(28)
O3'―H3'O**···**O4'	0.820(14)	1.945(23)	2.665(4)	146.0(28)
O5'―H5'O**···**O4'	0.820(21)	2.257(23)	2.698(3)	114.1(23)
O5―H5O**···**O4'^ i^	0.820(23)	2.104(35)	2.773(4)	138.5(32)
O6―H6O**···**O5'^ ii^	0.820(25)	1.871(26)	2.676(4)	166.5(30)
O5'―H5'O**···**O4^ iii^	0.820(21)	2.066(13)	2.762(3)	142.5(26)
O6'―H6'O**···**O5^ iv^	0.820(52)	1.893(52)	2.651(5)	153.2(51)
O2―H2O**···**O7^ v^	0.820(18)	1.815(19)	2.627(4)	169.9(20)
O7―H7O**···**O6^ vi^	0.820(38)	1.979(33)	2.769(5)	161.4(43)
O2'―H2'O**···**O7'^ vii^	0.820(49)	1.809(49)	2.626(5)	174.3(57)
O7'―H7'O**···**O6'^ viii^	0.820(18)	1.995(19)	2.789(6)	162.6(33)
C8―H8**···**O2^ vii^	0.980(6)	2.441(5)	3.124(5)	126.5(4)
**2**				
O3―H3O**···**O2	0.889(30)	2.128(30)	2.652(2)	117.0(24)
O5―H5O**···**O4	0.874(35)	2.187(27)	2.660(2)	113.6(26)
O6―H6O**···**O1^ix^	0.975(31)	1.848(30)	2.787(2)	160.8(27)
O5―H5O**···**O6^ i^	0.874(35)	2.312(28)	2.880(2)	122.7(27)
C2―H2**···**O5^ iii^	1.075(24)	2.386(24)	3.454(2)	172.3(18)
C13―H13**···**O6^ x^	1.018(23)	2.546(24)	3.452(2)	148.1(19)
C20―H201**···**O4^ xi^	0.965(30)	2.544(28)	3.464(3)	159.5(22)

Symmetry operators: (i) -1 + *x*, 1 + *y*, *z*; (ii) -1 + *x*, *y*, 1 + *z*; (iii) 1 + *x*, -1 + *y*, *z*; (iv) 1 + *x*, *y*, -1 + *z*; (v) 1 + *x*, *y*, *z*; (vi) *x*, 1 + *y*, -1 + *z*; (vii) -1 + *x*, *y*, *z*; (viii) *x*, -1 + *y*, 1 + *z*; (ix) *x*, -1 + *y*, *z*; (x) 1 - *x*, 0.5 + *y*, 2 – *z*; (xi) –*x* , 0.5 + *y*, 2 – *z*;

## Experimental

### General

Crystallization of the investigated compounds − dihydrokaempferol and lariciresinol − was carried out by the vapor diffusion method using organic solvents. Compound **1** (dihydrokaempferol) crystallizes in a triclinic system in space group P1 and compound **2** (lariciresinol) in a monoclinic system w space group P2_1_ with the unit cell consisting of two molecules. Crystal data and experimental details of both compounds are shown in Table 1. The overall view of all molecules with the atom numbering scheme is seen in Figure 2, the crystal packing diagram in Figure 3 and hydrogen bonding in Figure 4. The crystal structure of compound **1** was determined using data collected at room temperature on a CAD4 diffractometer with graphite monochromatized CuKα radiation (*λ* = 1.54184 Å). Maximum 2*θ* was 150º and the scan mode: *ω*/2*θ*. The diffraction data set for a crystal of compound **2** was collected with synchrotron radiation at EMBL beamline X13 (DESY Hamburg). Diffraction images were recorded using a Mar 165 mm CCD detector at 100 K. Compound **1** was crystallized separately from ethanol (**1a**) and from methanol (**1b**). The structure of **1** was determined with the molecules of both solvents trapped in the crystal lattices. Compound **2** was crystallized from methanol, but in this case the solvent did not trap in the unit cell of the crystal. For compound **2**, data was collected on a synchrotron (EMBL Hamburg) with two runs corresponding to low and high resolution. The high resolution run consisted of 90 images with oscillation 4º and the low resolution run consisted of 60 images with oscillation 6º. To avoid overloaded reflections, the exposure time for the low resolution run was ten times shorter than for the high resolution run. The diffraction data were processed with Denzo and scaled with Scalepack from the HKL program package [25].

An empirical absorption correction was applied for **1** by the use of the *ψ*-scan method (EAC program) [26,27]. All observed reflections with I > 0*σ*(I) were used to solve the structures by direct methods and to refine them by full matrix least-squares using *F^2^* [27,28]. Anisotropic thermal parameters were refined for all nonhydrogen atoms. Hydrogen atoms were found on the difference Fourier map and refined isotropically except for H atoms attached to carbon atoms of solvent in the crystal lattice of compound **1**. These hydrogen atoms were placed geometrically at idealized positions and set as riding with fixed thermal parameters equal to 1.33 times the equivalent isotropic thermal parameter of the parent-atom. The final calculation of compound **1a**, **1b**, **2** converged to R = 6.91%, R = 4.30%, R = 4.31% for 472, 454, 332 refined parameters and 2982, 2982, 4007 reflections with *I* ≥ 2*σ*(*I*), respectively. 

Data correction was carried out with the Enraf-Nonius SDP crystallographic computing package [26]; structure solution with SHELXS [28,29] and structure refinement with SHELXL [29,30]. The torsion angles and the dihedral angles between planes of aromatic rings of molecules were calculated by CSU [31]. CCDC 719360 (**1a**), CCDC 719361 (**1b**) and CCDC 719362 (**2**) contain the supplementary crystallographic data for this paper. These data can be obtained free of charge from the Cambridge Crystallographic Data Centre [23] via www.ccdc.cam.ac.uk/data_request/cif. 

## Conclusions

The investigated compounds are natural phenolic compounds. Dihydrokaempferol (**1**) is a member of the flavanones group, whilst lariciresinol (**2**) belongs to the lignans group − a group that consists of phenylpropane dimers enzymatically coupled through β-β-linkages between the propane chains. These phenolic compounds and their derivatives are antioxidants and should be investigated for their potential as antitumor agents [32,33]. The structural data presented in this work are a good basis for designing more biologically active inhibitors of lipid peroxidation and scavengers of superoxide radicals.
